# Intellectual disability associated with craniofacial dysmorphism due to *POLR3B* mutation and defect in spliceosomal machinery

**DOI:** 10.1186/s12920-022-01237-5

**Published:** 2022-04-18

**Authors:** Mostafa Saghi, Kolsoum InanlooRahatloo, Afagh Alavi, Kimia Kahrizi, Hossein Najmabadi

**Affiliations:** 1 Genetics Research Center, University of Social Welfare and Rehabilitation Sciences, Tehran, Iran; 2 School of Biology, College of Science, University of Tehran, Tehran, Iran

## Abstract

**Background:**

Intellectual disability (ID) is a clinically important disease and a most prevalent neurodevelopmental disorder. The etiology and pathogenesis of ID are poorly recognized. Exome sequencing revealed a homozygous missense mutation in the *POLR3B* gene in a consanguineous family with three Intellectual disability with craniofacial anomalies patients. *POLR3B* gene encoding the second largest subunit of RNA polymerase III.

**Methods:**

We performed RNA sequencing on blood samples to obtain insights into the biological pathways influenced by *POLR3B* mutation. We applied the results of our RNA-Seq analysis to several gene ontology programs such as ToppGene, Enrichr, KEGG.

**Results:**

A significant decrease in expression of several spliceosomal RNAs, ribosomal proteins, and transcription factors was detected in the affected, compared to unaffected, family members.

**Conclusions:**

We hypothesize that *POLR3B* mutation dysregulates the expression of some important transcription factors, ribosomal and spliceosomal genes, and impairments in protein synthesis and splicing mediated in part by transcription factors such as FOXC2 and GATA1 contribute to impaired neuronal function and concurrence of intellectual disability and craniofacial anomalies in our patients. Our study highlights the emerging role of the spliceosome and ribosomal proteins in intellectual disability.

**Supplementary Information:**

The online version contains supplementary material available at 10.1186/s12920-022-01237-5.

## Background

Intellectual disability (ID), a complex neurodevelopmental disorder, is defined as a notable impairment in cognitive and adaptive behavior before 18 years [1]. This condition, which affects approximately 1 to 3% of the general population, is a major health care problem of all developed countries. The etiology of ID can be divided into non-genetic and genetic insults. Non-genetic insults include a variety of environmental factors such as malnutrition, infection, trauma or head injury, and teratogens [2]. Most of these factors impose their effects during prenatal life [3]. Chromosomal abnormalities, dysregulation of genetic imprinting, and monogenic disease forms are significant contributors to ID [4].

Over the past 10 years, investigators have taken advantage of next-generation sequencing (NGS) technologies to identify many ID associated genes [5]. NGS is now being applied to analyze transcriptomes termed RNA-seq [6]. RNA-seq has played an important role in studying gene expression and identifying novel RNA species [7].

Although the number of genes responsible for ID increases rapidly, understanding the related processes is a challenge of basic and medical sciences. Abundant investigations have been applied to the study of the human brain based on the identification of genes implicated in ID [4]. Many of these genes in terms of modules interact together and have functional correlations that have been implicated in ID [8]. Some important molecular and biological mechanisms underlying ID have been recognized, including neurogenesis, synaptic structure and functions, immune system, and transcription and translation control [9].

A challenging area in intellectual disability is our poor understanding of the relationships among genes and how disruption of one gene affects that network and influences phenotype. Differential expression analysis is one method that can address this issue by deciphering the long lists of differentially expressed genes through combining them with other functional and ontological data [10].

RNA polymerase III (Pol III) is one of the three eukaryotic RNA polymerases. Pol III comprises 17 subunits with high conservation [11]. *POLR3A* and *POLR3B*, the two largest subunits of Pol III, encode the catalytic center of the enzyme [12]. Pol III is responsible for the synthesis of non-coding RNAs including 7SK RNA, Alu RNA, U6 RNA, H1 RNA, tRNA, 5S RNA, which are involved in cellular processes such as regulation of transcription, RNA processing, and translation [13]. Pol III plays a pivotal role in cellular processes and several studies have addressed the overall consequences of its dysfunction in mammalian cells [14, 15].

Mutations in *POLR3A* and *POLR3B* have been implicated in ID, which generally presents with 4H leukodystrophy. Recently, a study reported six unrelated individuals with *de novo* missense variants in *POLR3B gene and clinical presentation of* substantially different from POLR3-related leukodystrophy includes afferent ataxia, spasticity, variable intellectual disability and epilepsy, and predominantly demyelinating sensory-motor peripheral neuropathy [16].

This paper aims to identify differentially expressed genes and pathways in ID patients with a mutation in the *POLR3B* gene. We performed transcriptome analysis using RNA-sequencing on human blood cells carrying the *POLR3B* mutation.

## Methods

This study was conducted according to the declaration of Helsinki and with the approval of the ethics board of the University of Tehran. Participants consented to participate after being informed of the nature of the research.

### Subjects

**Fig. 1:**
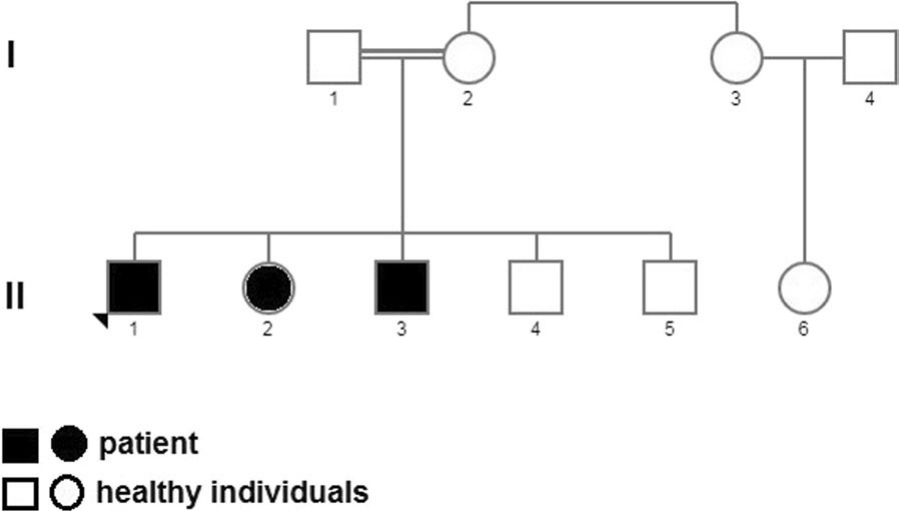
Pedigree of a family with more than two affected persons due to a homozygous missense mutation in *POLR3B*. II-1 is proband. II-2, II-3, II-4, II-5, II-6 and three healthy cousins (sex and age-matched) involved in this study

The consanguineous pedigree with three ID affected members was recruited (Fig. 1, Table 1). Transcriptome analysis was performed on two affected and six unaffected individuals of this family.

**Table 1:** Clinical features of subjects with mutation in POLR3B gene

		II:2	II:3
Gender		F	M
Age at examination		34	31
Postnatal Growth	HC (cm)	NA	NA
	Height (cm)	NA	NA
	BW (gr)	NA	NA
OFC (cm/SD)		55/ + 0.62	57.5/ + 1.67
Height (cm)		160	175
Weight (kg)		54	80
Facial appearance		long palpebral fissures, flat occiput, short philtrum, protrude ear and micrognathia	long palpebral fissures, flat occiput, short philtrum, protrude ear and micrognathia
Seizure	Time	–	–
	Medication response	–	–
Cognitive impairment		+	+
Intelligent quotient(IQ)		25	45
Spasticity		–	–
Walking		+	+
Hypotonia		–	–
Hypertonia		–	–
hyperreflexia		–	–
Stiff, rigid muscles, poor muscle function and paralysis	–	–	
Ataxia		–	–
Behaviour	Autistic behavior	+	+
	hyper activity	–	–
	ADHD	+	+
	aggression	–	–
	self injury	–	–
	Obsession	–	–
	Sterotypic movement	–	–
	Sterotypic speech	–	–
Eye	Strabismus	–	–
	Myopia	–	–
	Optic atrophy	NA	NA
	Retinal involvement	NA	NA
	Cataract	–	–
	Nystagmus	–	–
	Others	–	–
Ear	Hearing loss	–	–
Skeletal	Hand	–	–
	Foot	–	–
	Spine	–	–
Skin			
Speech			
Loss of bladder and bowel function		–	–
Gallbladder problems		–	–
MRI	Hypomyelination in MRI	NA	NA

+ , present; –, absent; MRI, magnetic resonance imaging; NA, not available; OFC, occipital frontal circumference; and SD: standard deviation

### RNA sequencing

Blood samples were collected from eight individuals mentioned above. Total RNA was extracted from the blood by the QIAamp RNA Blood Kit (Qiagen) following the manufacturer's instructions. RNA-seq libraries were generated using Illumina TruSeq stranded total RNA with ribo-zero globin Sample Prep kit. rRNA and globin RNA were exhausted using Illumina Globin Removal Mix. The RNA was fragmented into short pieces following the purification steps using RNA Fragmentation Reagents (Life Technologies). Under these conditions, fragment lengths range from 200 to 300 bp.

The Superscript II Reverse Transcriptase and random hexamers (Life Technologies) generated the first-strand complementary DNA. The second strand was synthesized using DNA Polymerase I and RNaseH. A single ‘A” base was added to the 3’ end, followed by ligation of the Illumina sequencing PE adapters. These products were then purified and enriched by polymerase chain reaction on the adapter-ligated cDNA with 2X Phusion DNA polymerase Master Mix (New England Biolabs). 10 µg of total RNA was used to generate index-inserted paired-end cDNA libraries. Finally, RNA samples were sequenced 100 bp (2 × 100) paired-end on Illumina HiSeq2500 (Macrogen).

### Data process

After obtaining the short reads, Sequence reads' quality from each sample was checked by FASTQC. Trimmomatic (v0.36) [17] was used to eliminate adaptors and low-quality bases. The ultrafast aligner Spliced Transcripts Alignment to a Reference software (STAR, v. 2.5.3) [18] was used to align all reads independently to a reference human genome assembly hg19 with the Illumina-supplied hg19 gene-model annotation file (gtf annotation). The mapped sequences were evaluated with FASTQC to ensure no artificial fragment representation. The output SAM files using SAMtools [19] were converted to BAM files, sorted by index. HTSeq-count (version 0.5.3p9) [20], a Python-based script, was used to calculate the number of aligned reads per gene.

### Differential expression analysis

To identify differentially expressed genes between the patients and the healthy controls, The DESeq2 (version 1.1.0, http://www.bioconductor.org/packages/release/bioc/html/ DESeq2.html) [21] package and Cufflinks (http://cufflinks.cbcb.umd.edu) [22] were used. Expression levels of all transcripts were normalized according to the fragments per kilobase of exon per million fragments mapped (FPKM) using Cufflinks. We used the filtering criteria, including p-value of ≤ 0.05 and fold change of ≥ 1.5 to categorize transcripts as significant differentially expressed genes (DEGs).

### Gene enrichment and pathway analysis

To assess the Functional annotation of the identified DEGs, pathway enrichment analysis for the DEGs (the intersection of DEGs from DESeq2 and Cufflinks) were conducted using several bioinformatics tools: ToppGene (https://toppgene.cchmc.org) [23], DAVID (https://david.ncifcrf.gov/) [24], Functional Enrichment analysis tool (FunRich v 3.1.3) [25], and Enrichr (http://amp.pharm.mssm.edu/Enrichr/) [26]. We selected pathways to adjust P-values of < 0.01 calculated by the Benjamini–Hochberg method implemented in these web tools.

### Protein–protein interaction analysis and transcriptional regulators

Brain protein–protein interactome (PPI) network of the proteins encoded by the DEGs was built using NetworkAnalyst [27]. NetworkAnalyst is a comprehensive online platform for visualization and gene expression data analysis is based on experimental studies and computational predictions. It was used to find crucial modules. Genes with enormous connections in the module are often hub genes, which may have essential functions.

Also, to find interactions, the JASPER database [28] for DEGs-TFs and miRTarBase v8.0 [29] database for Gene-miRNAs in NetworkAnalyst were applied to generating related networks.

### Comparison of our significant DEGs with genes identified in a mouse with mutation in Polr3b R103H causing Leukodystrophy

POLR3A and POLR3B protein sequences have great conservation between humans and mice. Bernard Brais et al. using mice models, have studied POLR3-related hypomyelinating leukodystrophy (POLR3-HLD). They found that *Polr3a* G672E homozygote mutation had no neurological deficits, and *Polr3b* R103H homozygote mutation was embryonically lethal.

Polr3a^G672E/G672E^/Polr3b^+/R103H^ double mutant mice were generated. Then, three affected mice were compared to three healthy mice using RNA-seq [30]. Here, using data from the Gene expression omnibus (GSE118739), we compared the DEGs in *Polr3b* mutated mouse and DEGs in our *POLR3B* mutated patients. DEGs were identified using DESeq2 (version 1.1.0) in the same way done for our gene list. Biomart was used to convert mouse gene IDs to their orthologous IDs in humans, then, DEGs in mice and DEGs in our ID patients were compared.

## Results

### POLR3B mutated family

There were eight participants in this study. Four of them are from a consanguineous family, including two patients and two controls. Figure 1 shows the family pedigree. Four other samples are healthy cousins matched by age and sex, which have added to more precise patient-control collation results.

Previously, Whole Exome Sequencing identified a homozygous missense mutation NM_018082.4:c.770C > A; p.(Thr257Lys)) in *POLR3B* gene [31]. The variant allele was completely absent in healthy controls in this study. *POLR3B* encodes one of the core components of RNA polymerase III, which transcripts small RNAs U2 and 5S rRNAs. This mutation caused severe intellectual disability, attention deficit, and autistic behavior with facial dysmorphism in three patients from first cousin healthy parents. Their facial appearances showed long palpebral fissures, flat occiput, short philtrum, protrude ear, and micrognathia.

Brain MRI of the oldest patient had hypomyelinating leukodystrophy. Cognitive status which was evaluated using WAIS-IV in three patients showed IQs of 25–40, in the range of severe ID. Table 1 details the phenotypes of the affected individuals.

### Differential expression analysis

**Fig. 2:**
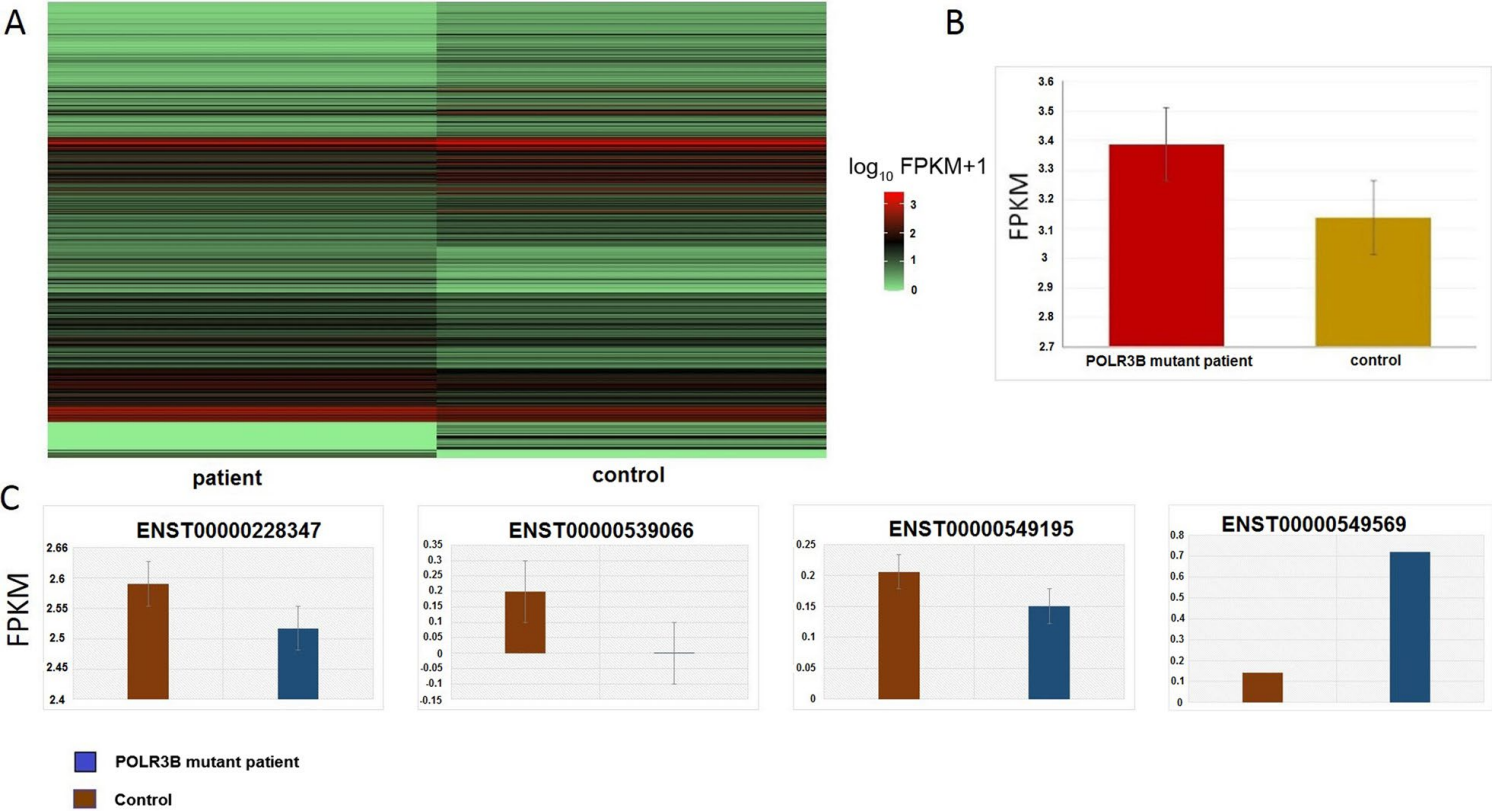
Differentially expressed genes between *POLR3B* mutant patients and 6 controls. **a** The heat map showing DEGs between *POLR3B* mutant patients vs controls. **b** Comparison of *POLR3B* expression in *POLR3B* mutant patients and controls. **c** Comparison of *POLR3B* isoform expression in *POLR3B* mutant patients and controls

Cuffdiff from the cufflinks package identified differential expressed genes between two patients and six healthy controls. We considered genes as DEGs with the parameter of p-value less than 0.05. We detected 532 differentially expressed genes, with 311 genes downregulated and 221 genes upregulated in ID patients compared to controls (Additional file 1: Table S1, Fig. 2a).

Among the downregulated genes, the expression value of 27 genes was zero in the patients; most of the genes are coding small nuclear RNAs (Table 2). Enrichment analysis for these 27 genes using the ToppGene site indicated pre-mRNA 5'-splice site binding (GO:0,030,627) as the primary Molecular Function (Table 3). From these 27 genes, 10 genes participate in spliceosome structure and mRNA splicing. For example, binding of U1 snRNA to the 5’ splice site is necessary for spliceosome assembly [32]. *RNU11* gene belongs to the snRNA class, and the mutation in this gene is associated with Microcephalic Osteodysplastic Primordial Dwarfism, Type I [33].

**Table 2:** Differentially expressed genes with no expression in POLR3B mutant patients or controls

Gene_id	Gene	Locus	Sample_1	Sample_2	Status	Value_1	Value_2	*p*_value
**Genes with no expression in patients**								
ENSG00000199347	RNU5E−1	1:11908151–11908271	Affected	Unaffected	OK	0	63.402	0.0099
ENSG00000200156	RNU5B-1	15:65304676–65304792	Affected	Unaffected	OK	0	77.1612	0.0121
ENSG00000207217	SNORA42	7:6009244–6059230	Affected	Unaffected	OK	0	33.804	0.0467
ENSG00000207392	SNORA20	6:159669056–159789749	Affected	Unaffected	OK	0	47.5036	0.04185
ENSG00000207501	RNVU1-14	1:145281115–145281462	Affected	Unaffected	OK	0	7.27258	0.0421
ENSG00000221676	RNU6ATAC	9:134164438–134164564	Affected	Unaffected	OK	0	51.8622	0.0016
ENSG00000222389	RNU2-28P	3:81489698–81762161	Affected	Unaffected	OK	0	4.97713	0.0419
ENSG00000225364	ATP6V0E1P1	5:132875378–132963634	Affected	Unaffected	OK	0	3.87561	0.03025
ENSG00000238151	MLLT10P1	20:30403122–30403384	Affected	Unaffected	OK	0	1.75103	0.0006
ENSG00000239576	COX6CP14	3:49724293–49786542	Affected	Unaffected	OK	0	5.87158	0.0393
ENSG00000240098	RN7SL351P	11:126202093–126278131	Affected	Unaffected	OK	0	2.05111	0.02335
ENSG00000240750	RN7SL559P	1:27,970343–28088696	Affected	Unaffected	OK	0	2.23239	0.0274
ENSG00000243243	AC073130.3	7:116209233–116508541	Affected	Unaffected	OK	0	1.40121	0.0043
ENSG00000243313	RN7SL285P	6:37819498–38154624	Affected	Unaffected	OK	0	3.5045	0.02865
ENSG00000244398	RP11-466H18.1	11:16778294–17053024	Affected	Unaffected	OK	0	3.40283	0.02655
ENSG00000244451	RPL34P21	11:130069836–130144811	Affected	Unaffected	OK	0	2.39474	0.0406
ENSG00000256148	RP11-809N8.5	11:73400486–73598189	Affected	Unaffected	OK	0	10.9554	0.0308
ENSG00000259235	RP11-605F22.2	15:48189036–48304078	Affected	Unaffected	OK	0	2.04009	0.0104
ENSG00000260035	CTD-2651B20.6	15:45092649–45201175	Affected	Unaffected	OK	0	2.9694	0.0221
ENSG00000267590	NDUFA3P1	19:44207546–44305046	Affected	Unaffected	OK	0	4.16525	0.0295
ENSG00000269987	RP3-430N8.11	22:30970676–30979395	Affected	Unaffected	OK	0	1.75279	0.0395
ENSG00000270103	RNU11	1:28648599–28648733	Affected	Unaffected	OK	0	25.5714	0.04965
ENSG00000275418	RP11-126O1.6	18:58659857–58660524	Affected	Unaffected	OK	0	2.3237	0.0001
ENSG00000276345	AC004556.1	KI270721.1:2584–11802	Affected	Unaffected	OK	0	1.93881	5.00E-05
ENSG00000277610	RNVU1-4	1:120913150–121052167	Affected	Unaffected	OK	0	8.82388	0.0395
ENSG00000278371	AL442127.1	13:106541672–106568164	Affected	Unaffected	OK	0	6.72073	0.0372
ENSG00000283125	RP11-299P2.2	18:63123345–63320128	Affected	Unaffected	OK	0	1.7566	0.03935
**Genes with no expression in controls**								
ENSG00000011052	NME1-NME2	17:51153535–51171747	Affected	Unaffected	OK	1.93848	0	0.00055
ENSG00000212579	SNORA40	6:35573584–35728583	Affected	Unaffected	OK	138.82	0	0.03135
ENSG00000229646	RP11-330A16.1	6:14597513–14599690	Affected	Unaffected	OK	20.8711	0	0.03115
ENSG00000229979	U82670.9	X:153052149–153052413	Affected	Unaffected	OK	1.64758	0	0.0001
ENSG00000233138	RP1-67K17.3	6:142748442–142945201	Affected	Unaffected	OK	2.16869	0	0.04335
ENSG00000248840	RP11-357G3.2	4:3293027–3439913	Affected	Unaffected	OK	1.88138	0	0.00625
ENSG00000280964	AL512384.1	6:25732496–25732827	Affected	Unaffected	OK	20.6383	0	0.0247

**Table 3:** The top molecular function, biological pathway and cellular component of the 27 genes with no expression in the patients (ToppGene)

	ID	Name	p Value	FDR B&H	FDR B&Y	Bonferroni
**GO: molecular function**						
1	GO:0030627	pre-mRNA 5'-splice site binding	6.55E−13	3.28E−12	7.48E−12	3.28E−12
2	GO:0036002	pre-mRNA binding	1.01E−10	2.52E−10	5.75E−10	5.03E−10
3	GO:0003723	RNA binding	1.08E−04	1.81E−04	4.12E−04	5.42E−04
4	GO:0030622	U4atac snRNA binding	8.24E−04	1.03E−03	2.35E−03	4.12E−03
5	GO:0017069	snRNA binding	1.11E−02	1.11E−02	2.53E−02	5.54E−02
**GO: biological process**						
1	GO:0022618	ribonucleoprotein complex assembly	1.49E−11	1.87E−10	6.81E−10	3.12E−10
2	GO:0071826	ribonucleoprotein complex subunit organization	1.78E−11	1.87E−10	6.81E−10	3.74E−10
3	GO:0000395	mRNA 5'-splice site recognition	5.34E−11	3.74E−10	1.36E−09	1.12E−09
4	GO:0000377	RNA splicing, via transesterification reactions with bulged adenosine as nucleophile	3.69E−10	1.24E−09	4.51E−09	7.76E−09
5	GO:0000398	mRNA splicing, via spliceosome	3.69E−10	1.24E−09	4.51E−09	7.76E−09
**GO: cellular component**						
1	GO:0097525	spliceosomal snRNP complex	2.46E−12	2.72E−11	9.19E−11	3.94E−11
2	GO:0030532	small nuclear ribonucleoprotein complex	3.47E−12	2.72E−11	9.19E−11	5.55E−11
3	GO:0120114	Sm-like protein family complex	5.10E−12	2.72E−11	9.19E−11	8.16E−11
4	GO:1990904	ribonucleoprotein complex	1.97E−08	7.87E−08	2.66E−07	3.15E−07
5	GO:0140513	nuclear protein-containing complex	4.77E−07	1.53E−06	5.16E−06	7.63E−06

Among the upregulated genes, the expression value of 7 genes was zero in the controls (Table 2). Between them, *NME1*-*NME2* was the only protein-coding gene (Table 2). NME1-NME2 are parts of the NME gene family with ten members. This locus represents naturally occurring read-through transcription between the neighboring *NME1* and *NME2* genes. Depending on tissue context, both have a crucial role in tumor progression and metastasis [34]. Recently, a study published in *Psychiatric Genetics* has represented a homozygous mutation in this gene can be associated with attention deficit hyperactivity disorder (ADHD) [35].

Our results revealed that the mutation didn’t change the expression level of *POLR3B*, so it presumably alters the function of its protein (Fig. 2b). *POLR3B* has four isoforms, and measurements show no significant difference between patients and controls (Fig. 2c).

**Fig. 3:**
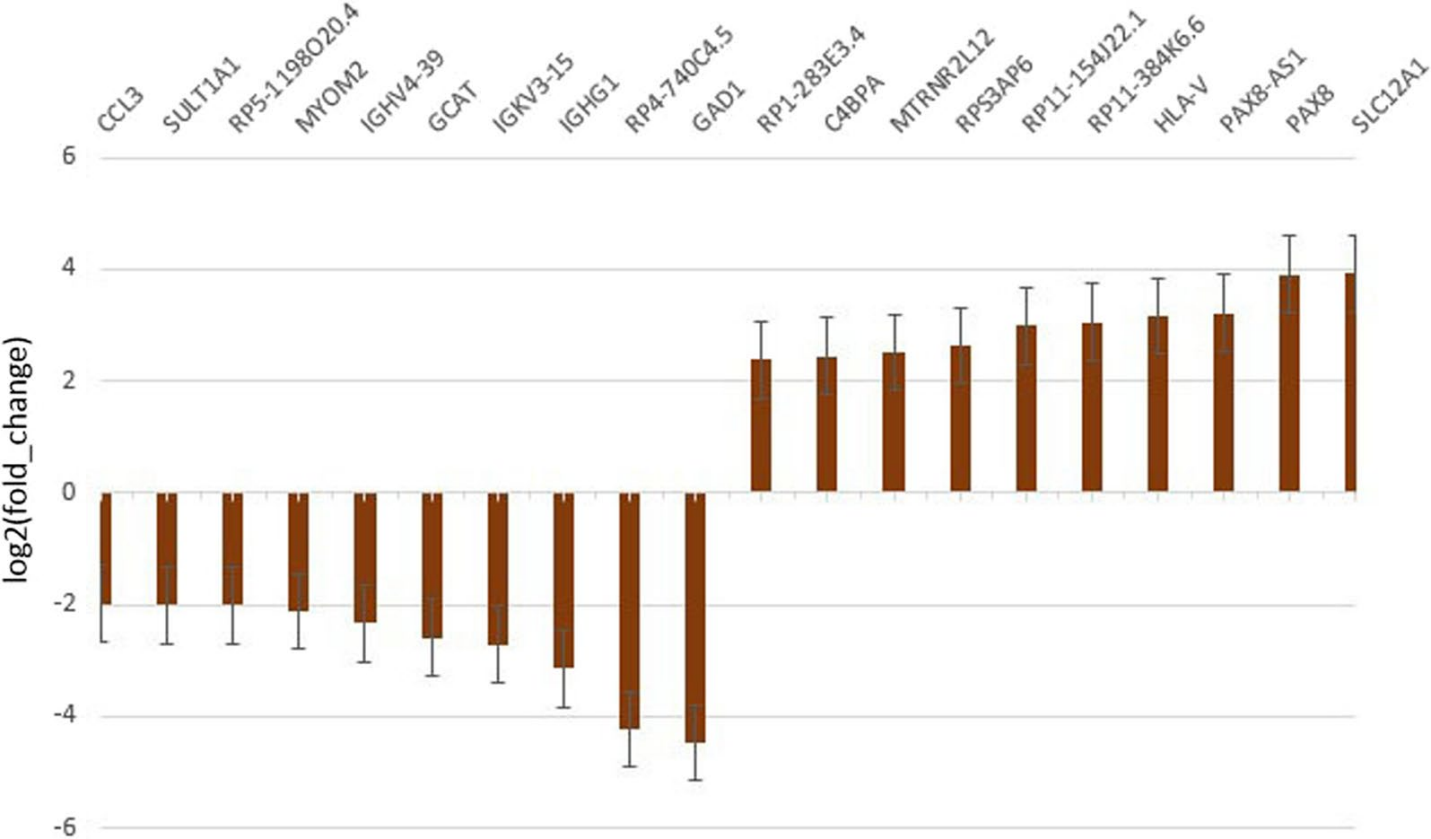
The top 10 down-regulated and up-regulated genes in *POLR3B* mutant patients vs controls

The top 10 down-regulated genes in the patients are listed in Table 4 and Fig. 3. Of these, the *SLC12A1* gene had the greatest fold change. *SLC12A1* encodes solute carrier family 12 members 1 protein and is implicated in ID in the literature [36]. The top 10 up-regulated genes in the patients are listed in Table 4 and Fig. 3. The *GAD1* gene with the greatest fold change was reported as a causative gene associated with syndromic developmental and epileptic encephalopathy [37]. Among DEGs, 30 genes were reported as an ID gene [38] (Table 5). Of these, 21 genes were down-regulated in the patients.

**Table 4:** The top 10 down-regulated and up-regulated genes in POLR3B mutant patients

Gene symbol	Foldchange	p_value	Function	Top biological pathway
**Up-regulated**				
SLC12A1	15.2	5.00E−05	SLC12A1 (solute carrier family 12 member 1)	Cation-coupled Chloride cotransporters
PAX8	15	0.00095	PAX8 (paired box 8)	DNA-binding transcription factor activity and RNA polymerase II core promoter sequence-specific DNA binding
PAX8-AS1	9	0.01	PAX8-AS1 (PAX8 antisense RNA 1)	A potential regulator of PAX8
HLA-V	8	0.03295	HLA-V (major histocompatibility complex, class I, V (pseudogene))	Pseudogene
RP11-384K6.6	8	5.00E−05		Non-coding RNA
RP11-154J22.1	7.9	0.02655		Non-coding RNA
RPS3AP6	6	0.00385		RPS3A Pseudogene 6
MTRNR2L12	5.6	0.00435	MTRNR2L12 (MT-RNR2 like 12)	Pseudogene
C4BPA	5.4	0.0006	Complement Component 4 Binding Protein Alpha	RNA binding
RP1-283E3.4	5.2	0.03535		Pseudogene
**Down-regulated**				
GAD1	22.1	0.0154	Glutamate decarboxylase 1	Pyridoxal phosphate binding
RP4-740C4.5	18.7	0.0368		pseudogene
IGHG1	8.9	5.00E−05	Immunoglobulin heavy constant gamma 1 (G1m marker)	Antigen binding
IGKV3-15	6.59	5.00E−05	Immunoglobulin kappa variable 3–15	Antigen binding
GCAT	6	5.00E−05	Glycine C-acetyltransferase	Pyridoxal phosphate binding
IGHV4-39	5	5.00E−05	Immunoglobulin heavy variable 4–39	Antigen binding
MYOM2	4	0.00035	Myomesin 2	Structural constituent of muscle
RP5-1198O20.4	4	0.0016		LincRNA
SULT1A1	4	5.00E−05	Sulfotransferase family 1A member 1	Sulfotransferase activity and flavonol 3-sulfotransferase activity
CCL3	3.9	0.00285	C–C motif chemokine ligand 3	Immune system, chemokine activity

**Table 5:** Dysregulated ID genes in POLR3B mutated patients

Gene symbol	log 2FoldChange	p value	Gene name	Model_of_inheritance	Phenotypes
ADGRG1	0.771931	0.0163	Adhesion G Protein-Coupled Receptor G1	BIALLELIC, autosomal or pseudoautosomal	Polymicrogyria, bilateral frontoparietal, 606,854
ADPRHL2	− 0.549797	0.04495	ADP-Ribosylserine Hydrolase	BIALLELIC, autosomal or pseudoautosomal	Developmental regression;Seizures;Ataxia;Intellectual disability
ARV1	0.998172	0.02185	ARV1	BIALLELIC, autosomal or pseudoautosomal	Epileptic encephalopathy, early infantile, 38
ATP6V1A	− 0.605652	0.03905	ATPase H + Transporting V1 Subunit A	MONOALLELIC, autosomal or pseudoautosomal, imprinted status unknown	Epileptic encephalopathy, infantile or early childhood,
ATP8B1	1.54362	0.0248	ATPase Phospholipid Transporting 8B1	BIALLELIC, autosomal or pseudoautosomal	Cholestasis, progressive familial intrahepatic 1,
CEP290	0.965876	0.0449	Centrosomal Protein 290	BIALLELIC, autosomal or pseudoautosomal	Joubert syndrome 5, 610188Senior-Loken syndrome 6
CLCN4	− 0.989201	0.0033	Chloride Voltage-Gated Channel 4	X-LINKED: hemizygous mutation in males	Mental retardation, X-linked 49–15 300,114
COX7B	1.38666	0.0017	Cytochrome C Oxidase Subunit 7B	X-LINKED: hemizygous mutation in males	Gene2Phenotype confirmed gene with ID HPO
CYP27A1	0.882676	0.016	Cytochrome P450 Family 27 Subfamily A Member 1	Other—please specify in evaluation comments	Cerebrotendinous xanthomatosis
DDX11	2.25969	0.02515	DEAD/H-Box Helicase 11	BIALLELIC, autosomal or pseudoautosomal	WARSAW BREAKAGE SYNDROME (WBRS)
EEF1B2	1.08279	0.04285	Eukaryotic Translation Elongation Factor 1 Beta 2	BIALLELIC, autosomal or pseudoautosomal	AUTOSOMAL RECESSIVE MENTAL RETARDATION
EZH2	0.977038	0.03085	Enhancer Of Zeste 2 Polycomb Repressive Complex 2 Subunit	MONOALLELIC, autosomal or pseudoautosomal, NOT imprinted	WEAVER SYNDROME 2
FHL1	− 0.673851	0.01625	Four And A Half LIM Domains 1	X-LINKED: hemizygous mutation in males, biallelic mutations in females	Scapuloperoneal myopathy
GATM	1.02915	0.04055	Glycine Amidinotransferase	BIALLELIC, autosomal or pseudoautosomal	Cerebral creatine deficiency syndrome 3,
HCFC1	0.832009	0.0466	Host Cell Factor C1	X-LINKED: hemizygous mutation in males, biallelic mutations in females	Mental retardation, X-linked 3, 309,541;MENTAL RETARDATION, X-LINKED 3; MRX3
HPRT1	1.1809	0.01315	Hypoxanthine Phosphoribosyltransferase 1	X-LINKED: hemizygous mutation in males, biallelic mutations in females	Lesch-Nyhan syndrome, 300322HPRT-related gout, 300,323;GOUT HPRT-RELATED (GOUT-HPRT)
IRF2BPL	− 0.805618	0.00795	Interferon Regulatory Factor 2 Binding Protein Like	MONOALLELIC, autosomal or pseudoautosomal, imprinted status unknown	Neurodevelopmental disorder with regression, abnormal movements, loss of speech, and seizures
ISPD	1.6776	0.01625	CDP-L-Ribitol Pyrophosphorylase A	BIALLELIC, autosomal or pseudoautosomal	Muscular dystrophy-dystroglycanopathy
KIF11	0.851313	0.0331	Kinesin Family Member 11	MONOALLELIC, autosomal or pseudoautosomal, NOT imprinted	AUTOSOMAL-DOMINANT MICROCEPHALY
KLF1	0.335299	0.3098	Kruppel Like Factor 1	MONOALLELIC, autosomal or pseudoautosomal, imprinted status unknown	Dyserythropoietic anemia, congenital, type IV,
NDUFS4	0.885125	0.03065	NADH:Ubiquinone Oxidoreductase Subunit S4	BIALLELIC, autosomal or pseudoautosomal	Leigh syndrome
PAX8	3.91102	0.00095	Paired Box 8	MONOALLELIC, autosomal or pseudoautosomal, NOT imprinted	Hypothyroidism, congenital, due to thyroid dysgenesis or hypoplasia
RBBP8	0.802837	0.00905	RB Binding Protein 8, Endonuclease	BIALLELIC, autosomal or pseudoautosomal	Jawad syndrome, 251,255;Microcephaly with mental retardation
SAMHD1	0.607404	0.0341	SAM And HD Domain Containing Deoxynucleoside Triphosphate Triphosphohydrolase 1	BIALLELIC, autosomal or pseudoautosomal	AICARDI-GOUTIERES SYNDROME
SMAD3	0.508352	0.0497	SMAD Family Member 3	MONOALLELIC, autosomal or pseudoautosomal, imprinted status unknown	Loeys-Dietz syndrome, type 3, 613,795
SPTAN1	0.73025	0.0173	Spectrin Alpha, Non-Erythrocytic 1	MONOALLELIC, autosomal or pseudoautosomal, NOT imprinted	EPILEPTIC ENCEPHALOPATHY EARLY INFANTILE TYPE 5 (EIEE5)
TSEN34	− 0.788859	0.00405	TRNA Splicing Endonuclease Subunit 34	BIALLELIC, autosomal or pseudoautosomal	PONTOCEREBELLAR HYPOPLASIA TYPE 2 AND TYPE 4
TUBB2A	− 1.36357	0.00035	Tubulin Beta 2A Class IIa	MONOALLELIC, autosomal or pseudoautosomal, NOT imprinted	CORTICAL DYSPLASIA, COMPLEX, WITH OTHER BRAIN MALFORMATIONS 5
VRK1	0.887767	0.0193	VRK Serine/Threonine Kinase 1	BIALLELIC, autosomal or pseudoautosomal	Pontocerebellar hypoplasia type 1A, 607,596;PONTOCEREBELLAR HYPOPLASIA TYPE 1
WDR45	− 0.120744	0.7569	WD Repeat Domain 45	BIALLELIC, autosomal or pseudoautosomal	AUTOSOMAL RECESSIVE MENTAL RETARDATION

### Pathway analysis

To assess the biological process and significant molecular mechanisms underlying the pathogenesis of ID, we analyzed the DEGs by ToppFun application of ToppGene suite in terms of molecular function, cellular component, biological process, and biological pathway. The analyses showed spliceosomal snRNP assembly and innate immune response were involved as the main biological processes (Table 6). The molecular functions and cellular components encoded by the DEGs were significantly related to the ribosome and its subunits, spliceosomal snRNP complex, and Nonsense Mediated Decay (NMD) (Table 6).

**Table 6:** Differentially expressed pathways in POLR3B mutant patients using ToppGene

Category	ID	p-value	q-value Bonferroni	Hit count in query list	Hit count in genome	Hit in query list
**KEGG pathway**						
	Ribosome	2.12E−10	4.83E−07	16	88	RPL26,RPL27,RPL31,RPL34,RPL39,RPL41,RPS16,RPS17,RPS21,RPS24,RPS27,RPS27A,RPL35,RPL7,RSL24D1,RPL9
	Nonsense Mediated Decay (NMD) independent of the Exon Junction Complex (EJC)	1.53E−09	3.47E−06	16	100	RPL26,RPL27,RPL31,RPL34,RPL39,RPL41,RPS16,RPS17,RPS21,RPS24,RPS27,RPS27A,LOC101929876,RPL35,RPL7,RPL9
	rRNA processing	2.71E−05	6.17E−02	16	203	RPL26,RPL27,RPL31,RPL34,RPL39,RPL41,RPS16,RPS17,RPS21,RPS24,RPS27,RPS27A,LOC101929876,RPL35,RPL7,RPL9
	Spliceosomal snRNP assembly	1.44E−06	7.61E−03	9	53	RNU6ATAC,RNU5E-1,RNU5B-1,RNU4-2,RNU4-1,SMN2,SNRPD2,SNRPG,STRAP
	Innate Immune System	2.14E−06	4.86E−03	58	1312	TRAF3,ABCA13,RPS27A,LY96,CHI3L1,ADCY9,S100A8,TUBB4B,MGST1,MAVS,MME,MMP8,MMP9,HSP90AA1,ADGRG3,ANGPT1,DEFA1B,ITLN1,CEP290,ICAM3,GMFG,OLFM4,PYCARD,IRS2,VAMP8,IL3RA,IL5RA,CLEC4C,CXCR1,PROS1,CYBA,ATP6V1A,ATP6V0B,SPTAN1,ITGA2B,ITGB3,CLEC12A,CFD,VNN1,NFKBIA,C3AR1,C4BPA,CRISPLD2,DUSP1,CAMP,DUSP2,RAB44,RNASE2,CD3G,RNASE6,ATP6V0D1,CD14,P2RX7,GZMM,CD180,HBB,CDC34,HERC5
**Molecular function**						
GO:0,003,735	Structural constituent of ribosome	1.82E−08	1.93E−05	19	183	RPL26,RPL27,RPL31,RPL34,RPL39,RPL41,RPS16,RPS17,RPS21,RPS24,RPS27,RPS27A,MRPL35,MRPL22,MRPS33,RPL35,RPL7,RSL24D1,RPL9
GO:0,004,601	Peroxidase activity	2.93E−05	3.10E−02	8	57	MGST1,PRDX5,ALOX5AP,PTGS1,HBA2,HBM,HBB,HBQ1
GO:0,016,209	Antioxidant activity	2.99E−05	3.17E−02	10	92	S100A8,MGST1,PRDX5,ALOX5AP,SRXN1,PTGS1,HBA2,HBM,HBB,HBQ1
GO:0,031,720	Haptoglobin binding	4.09E−05	4.33E−02	4	10	HBA2,HBM,HBB,HBQ1
**Biological process**						
GO:0,000,387	Spliceosomal snRNP assembly	1.44E−06	7.61E−03	9	53	RNU6ATAC,RNU5E-1,RNU5B-1,RNU4-2,RNU4-1,SMN2,SNRPD2,SNRPG,STRAP
GO:0,045,087	Innate immune response	1.85E−06	9.78E−03	46	1045	TRAF3,RPL39,HLA-DQA2,HLA-DRB5,ZNF683,LY96,S100A8,TUBB4B,MAVS,IFITM3,FES,CCL3,HSP90AA1,ANXA1,DEFA1B,ICAM3,SAMHD1,RAB20,IFI27,NLRP2,NLRP6,RSAD2,PIK3R6,PYCARD,CD24,XAF1,VAMP8,CLEC4C,MX1,CYBA,IFIT5,TRIM14,NDUFS4,CFD,GBP4,VNN1,C4BPA,APOBEC3C,IGHV4-39,CAMP,RNASE2,RNASE6,CD14,GZMM,CD180,HERC5
GO:0,022,613	Ribonucleoprotein complex biogenesis	4.62E−06	2.44E−02	27	484	RPL26,RPL27,RPS16,RPS17,RPS21,RPS24,RPS27,RNU6ATAC,RNU11,RNVU1-14,RNVU1-4,RNU5E-1,RNU5B-1,RNU4-2,RNU4-1,HSP90AA1,EIF2S3B,NIFK,MRPL22,SMN2,SNRPD2,SNRPG,RRP1B,STRAP,RPL35,RPL7,RSL24D1
GO:0,006,413	Translational initiation	5.12E−06	2.70E−02	16	199	RPL26,RPL27,RPL31,RPL34,RPL39,RPL41,RPS16,RPS17,RPS21,RPS24,RPS27,RPS27A,EIF2S3B,RPL35,RPL7,RPL9
GO:0,045,291	mRNA trans splicing, SL addition	3.66E−05	1.93E−01	4	10	RNU5E-1,RNU5B-1,RNU4-2,RNU4-1
GO:0,000,353	Formation of quadruple SL/U4/U5/U6 snRNP	3.66E−05	1.93E−01	4	10	RNU5E-1,RNU5B-1,RNU4-2,RNU4-1
GO:0,000,365	mRNA trans splicing, via spliceosome	3.66E−05	1.93E−01	4	10	RNU5E-1,RNU5B-1,RNU4-2,RNU4-1
GO:0,000,244	Spliceosomal tri-snRNP complex assembly	1.51E−04	7.95E−01	5	25	RNU6ATAC,RNU5E-1,RNU5B-1,RNU4-2,RNU4-1
GO:0,000,395	mRNA 5'-splice site recognition	1.47E−03	1.00E + 00	4	24	RNU6ATAC,RNU11,RNVU1-14,RNVU1-4
**Cellular component**						
GO:0,005,840	Ribosome	8.00E−08	5.29E−05	22	269	RPL26,RPL27,RPL31,RPL34,RPL39,RPL41,RPS16,RPS17,RPS21,RPS24,RPS27,RPS27A,MRPL35,MRPL22,MRPS33,LARP4,AURKAIP1,RPL35,HBA2,RPL7,RSL24D1,RPL9
GO:1,990,904	Ribonucleoprotein complex	4.88E−05	3.22E−02	34	768	RPL26,RPL27,RPL31,RPL34,RPL39,RPL41,RPS16,RPS17,RPS21,RPS24,RPS27,RPS27A,MRPL35,RNU6ATAC,RNU11,RNVU1-14,RNVU1-4,RNU5E-1,RNU5B-1,RNU4-2,RNU4-1,VBP1,MRPL22,MRPS33,SNRPD2,SNRPG,MKRN3,RRP1B,LARP4,RPL35,HBA2,RPL7,RSL24D1,RPL9
GO:0,034,719	SMN-Sm protein complex	6.07E−04	4.01E−01	4	19	SMN2,SNRPD2,SNRPG,STRAP
GO:0,005,925	Focal adhesion	8.12E−04	5.37E−01	20	424	RPL27,RPL31,RPS16,RPS17,FES,MME,FHL1,ANXA1,SNTB1,CYBA,GAK,ITGA2B,ITGB3,MRC2,DPP4,RDX,AFAP1,CD151,RPL7,RPL9
GO:0,062,023	Collagen-containing extracellular matrix	1.07E−03	7.09E−01	22	498	ACHE,PF4,F13A1,S100A8,FBN2,CLC,MMP8,MMP9,PLOD1,SDC2,HSP90AA1,COL1A2,ANGPT1,ANXA1,DEFA1B,ITLN1,CCDC80,MMRN1,MXRA7,CD151,EGFL7,HCFC1
GO:0,005,687	U4 snRNP	3.72E−03	1.00E + 00	6	70	RNU5E-1,RNU5B-1,RNU4-2,RNU4-1,SNRPD2,SNRPG
GO:0,097,525	Spliceosomal snRNP complex	4.01E−03	1.00E + 00	10	173	RNU6ATAC,RNU11,RNVU1-14,RNVU1-4,RNU5E-1,RNU5B-1,RNU4-2,RNU4-1,SNRPD2,SNRPG

“Hit Count in Query List” is the number of genes in our list of differentially expressed genes which involved in a specific pathway and “Hit Count in Genome” is the number of all genes involved in this specific pathway

The most striking pathways related to intellectual disability were "immune system", "translation", "spliceosomal snRNP assembly", "Nonsense Mediated Decay" (Table 6).

### Protein–protein interaction analysis

**Fig. 4:**
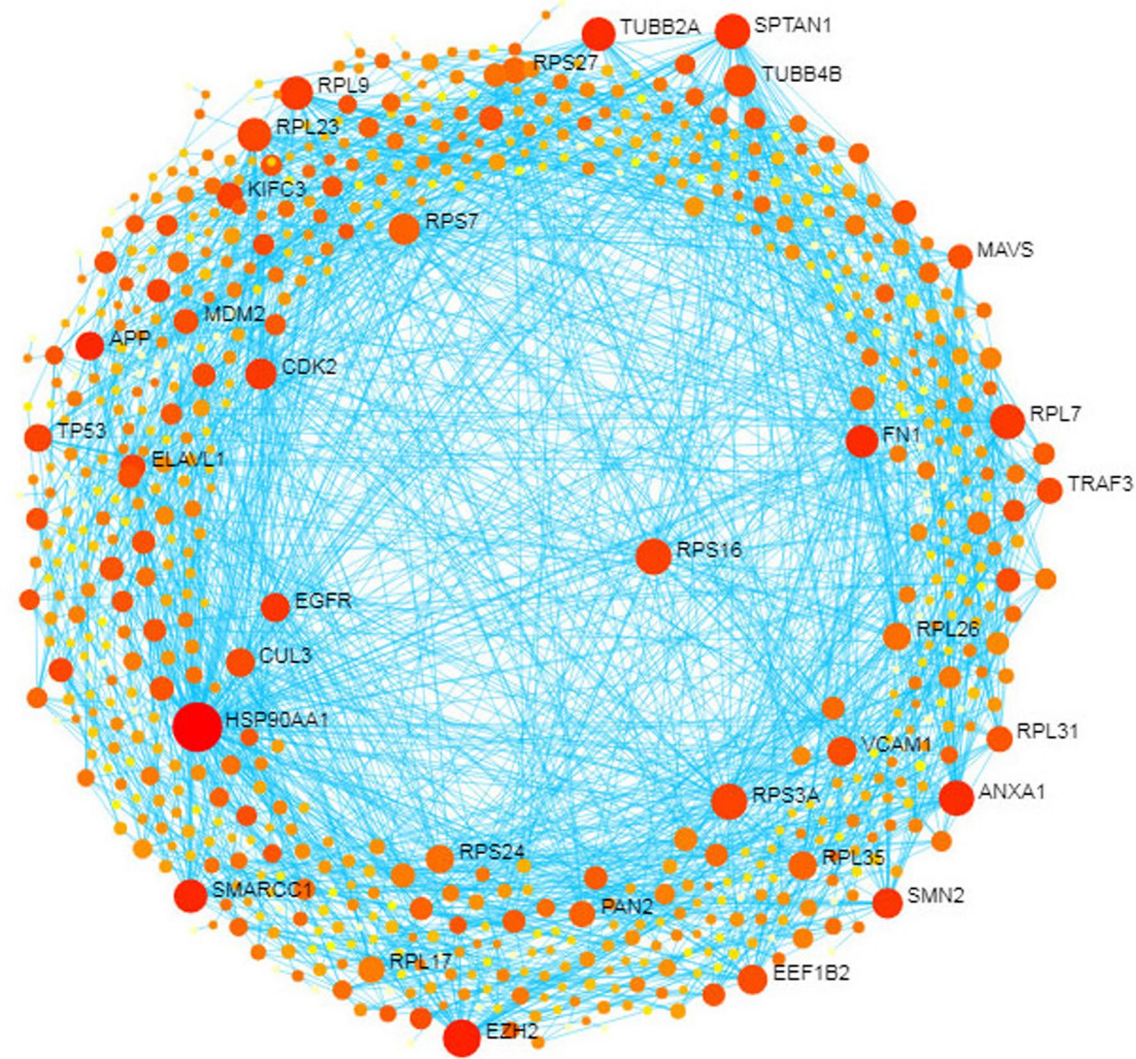
protein–protein interactions (PPI) of the DEGs. Nodes and edges represented by colored circles and arrows respectively. The big circle nodes are the hub proteins

We used tissue-specific (cortex) protein–protein interactome data to construct protein–protein interaction (PPI) network. Several subnetworks around the DEGs were achieved, the first subnetwork had 2495 nodes, and 4169 edges contained 288 seed nodes. Then, a minimum network was applied to reconstruct a subnetwork with 749 nodes, 2044 edges, and 288 seeds. Network analyst software was applied to visualize the interaction network (Fig. 4). The degree-based topological analysis with force atlas layout showed 34 Hub genes. Additional file 1: Table S2 lists the details of the Hub proteins.

### Transcriptional regulators

**Fig. 5:**
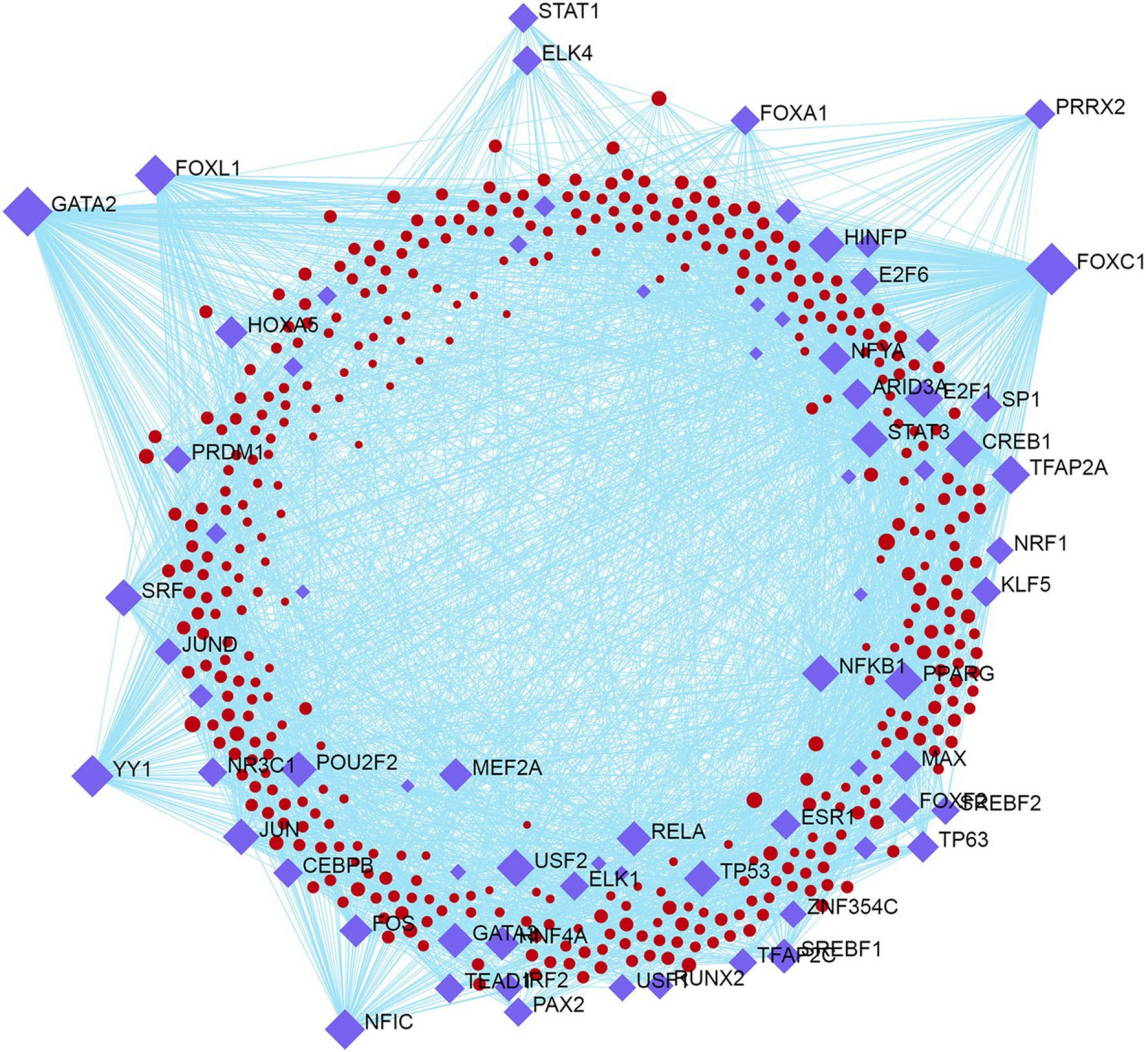
Transcriptional regulators of DEGs and miRNA. Transcriptional regulators of DEGs. Diamonds represent TFs and red circles show nodes, they are related by arrows

We constructed a TF-Genes network-based interaction using the JASPER database. We applied the minimum network option on subnetworks to attain a unique network and filtered the result by brain tissue. The reconstructed subnetwork had 485 nodes, 3218 edges, and 411 seed nodes (Fig. 5). Transcription factors with binding sites for greater number of DEGs were FOXC1, GATA2, FOXL1, YY1, NF1C, STAT1, and ELK4 (Table 7).

**Table 7:** Transcription factor binding site

Gene	Description	Degree	Clinical significance	Reference
Transcription factors using JASPER database				
FOXC1	Forkhead Box C1	239	WAGER syndrome	Genecards
			Microcephaly intellectual disability	Genecards
				[28, 29]
GATA2	GATA Binding Protein 2	200	Intellectual Disability	[34]
			Myelodysplastic syndrome	[31–33]
YY1	YIN-YANG-1	113	rett syndrome	Genecards
			Intellectual Disability	[37]
FOXL1	Forkhead Box L1	103	autism	[43]
NFIC	Nuclear Factor I C	96	Bipolar disorder	[38–40]
			Schizophrenia	
PPARG	Peroxisome Proliferator Activated Receptor Gamma	85	Alzheimer disease	Genecards
E2F1	E2F Transcription Factor 1	85	Epilepsy	Genecards
PRRG4	Proline Rich And Gla Domain 4	33	WAGER syndrome	[44]
hsa-mir-92a-3p	microRNA	29	Autoimmune disease of CNS	Genecards
			Nervous system disease	Genecards
			Schizophrenia	[45]
			Autism	[46]

Also, we investigated the relation of Gene-miRNAs using miRTarBase v8.0 database, which experimentally validated miRNAs-Gene interaction data. We attained a network with more than 2000 nodes, so we used a minimum network option and considered nodes with at least 10 degrees. After filtering, a subnetwork with 102 nodes, 465 edges and 33 seeds were built. PRRG4 and mir-92a-3p were the crucial node and miRNA, respectively.

### Comparison of our significant degs to genes identified in a mouse with an R103H mutation in Polr3b causing Leukodystrophy

From 255 DEGs in mice, 147 genes have a homolog in humans. Among these 147 genes, 5 were shared between our *POLR3B* mutated patients and *Polr3b* mutated mice (MYL4, RAB44, LY6G6E, TRAF5, CKM). Tumor necrosis factor receptor-associated factor 5 (TRAF5) interacts with downstream effectors, including tumor necrosis factor (TNF) and interleukin-1 receptor/Toll-like receptor. TRAF5 plays key role in regulating several signaling pathways such as Nod-like receptor pathway and Akt/FoxO1 signaling pathway. It has been found neuronal apoptosis level, blood–brain barrier (BBB) degradation, and inflammatory response reduced in TRAF5 Knockout Mice. Also, TRAF5 protein expression significantly increased in ischemic brains [39].

## Discussion

The advent of next-generation sequencing technologies has detected a large number of causative genes in ID. Studies of transcriptome changes in ID patients compared to healthy controls, are limited due to the difficulties in accessing tissues, here we performed a comprehensive transcriptome analysis of total RNA extracted from the blood from members of a family affected by a recessive mutation *POLR3B*. Our data's most significant differentially expressed pathways between patients and healthy controls were Ribosome, Nonsense-mediated decay, spliceosomal snRNP assembly, immune system.

Our results showed that numerous spliceosomal genes were significantly dysregulated in *POLR3B* mutant patients. The spliceosome is a large protein-RNA complex that removes introns from nuclear pre-mRNA. Researchers have revealed mutations in pre-mRNA splicing factor genes causes craniofacial anomalies [40]. Along the same lines, studies have shown mutation in components of spliceosome causes concurrence of ID, short stature, poor speech, and craniofacial anomalies [41]. Recently, Lee and colleagues have shown X-linked ID causative mutations in the FAM50A gene dysregulate the expression of spliceosomal RNAs and transcripts involved in neurodevelopment [42]. Furthermore, mutations in subunits of RNA polymerase III (POLR1D and POLR1C) have been identified in Treacher Collins syndrome (TCS), which is a malformation craniofacial disorder [43, 44]. Here, we detected a significant decrease expression of some spliceosomal RNAs (Table 2) in our ID patients. Clinical features of our patients (Table 1) shows that they have severe craniofacial anomalies. Finally, we predict that *POLR3B* mutation in our patients dysregulated expression of splicing factor genes and caused Intellectual disability with craniofacial anomalies in our patients.

Downregulation of numerous ribosomal proteins was also observed in *POLR3B* mutant patients, and ribosome was one of the most significant pathways dysregulated in the current study. Twenty-two ribosomal proteins include ribosomal S, L, and M subunits downregulated in our patients. RNA polymerase III synthesizes transfer and small ribosomal RNAs in eukaryotes. Ribosome biogenesis plays key role in regulating protein synthesis capacity in different tissues[45]. Previous studies have shown deficiency of ribosomal proteins may cause a reduction in rRNA synthesis and vice versa [46, 47]. Therefore, it seems that the downregulation of ribosomal proteins in our patients is due to *POLR3B* deficiency and a reduction of rRNA synthesis. In the literature, RPL10 mutations have been reported to cause neurodevelopmental disorders with the clinical spectrum from autism to syndromic ID [48, 49]. Studies have shown that several ribosomal genes were downregulated in the hippocampus of Alzheimer patients (AD)[50]. Recently, scientists recommended that ribosomal dysfunction in peripheral blood might be related to prodrome and progression of AD [51]. Therefore, downregulation of ribosomal proteins in our patients may disrupt protein synthesis and contribute to cognitive impairment.

Our DEGs include several cell adhesion molecules (CAMs) and immune system genes such as ICAM3, SELP, CLDN5, CD274, CD8B, CD8A, SDC2. CAMs play important roles in the nervous system. They control the interaction of neurons and glia, synapse formation and neurite outgrowth [52]. Three genome –wide association studies (GWAS) demonstrated aberrant CAM molecules are associated with neurological disorders, including schizophrenia and bipolar disorder[53]. Several ID and neurodevelopmental disease causative mutations in different CAMs such as L1CAM and ICAM3 have been demonstrated in different studies [54].

This study also identified the potential TFs using the topological analysis of protein–protein interactions (Table 7). They include FOXC1, GATA2, YY1, FOXL1, NFIC, PPARG, and E2F1. FOXC1 deletion or duplication can lead to cerebellar and posterior fossa malformations [55]. Also, two case report studies have also shown that ring chromosome 6 encompassing FOXC1 is associated with intellectual disability, short stature, and multiple facial dysmorphisms [56, 57]. GATA2 expression in posterior diencephalon-midbrain is crucial to GABAergic neuron development, migration, and regulation of neuron-specific gene expression [58]. Whole-Exome Sequencing revealed a mutation in GATA2 causes a rare Syndromic Congenital Neutropenia With Intellectual Disability [59]. FOXl1 is another member of Forkhead box (FOX) proteins whose dysregulation activates the Wnt/b-catenin pathway. [60] YY1 controls brain development, proliferation, and survival of neural progenitor cells (NPCs) By regulating many metabolic pathways and protein translation [61]. YY1 deletions and point mutations lead to syndromic ID with a wide variety of phenotypic features, including cognitive impairment, behavioral alterations, intrauterine growth restriction because of transcriptional and chromatin dysfunction [62]. *PRRG4* is located in the 11p13 region, relevant to WAGR syndrome. Toshiyuki Yamamoto et al. suggested haploinsufficiency of *PAX6* or *PRRG4* caused severe developmental delay and autistic behaviors of WAGR syndrome [63].

We investigated miRNAs-Gene interaction using miRTarBase v8.0 database. As a result, PRRG4 and mir-92a-3p were the crucial node and miRNA. mir-92a-3p has an association with synaptic structure and function. Also, it was identified as a biomarker in peripheral blood for schizophrenia [64]. In addition, a study on gene regulatory associated with autism in the Chinese population showed mir-92a-3p dysregulation in the peripheral blood of patients [65].

In the current study, we have used blood transcriptome to identify differentially expressed genes and pathways in *POLR3B* mutant patients. Since brain tissues were not available, we performed RNA sequencing on patients’ blood samples. Several studies have shown great similarity between blood and brain transcriptome. Therefore, blood is considered good an alternate. Our results have shown that mutation in *POLR3B* gene dysregulated the expression of some important transcription factors and spliceosome genes. DEGs are involved in some important biological processes such as spliceosome assembly, ribosome, and NMD.

## The limitations of this study

A single family with a single variant was analysed. The analysis is in blood derived RNA rather than brain. A small sample size of affected individuals were analysed thus a high number of false positive findings are expected. Inability to independently replicate the key findings using an alternative analysis of RNA expression such as quantitative real-time PCR.

## Conclusion

We hypothesize that *POLR3B* mutation dysregulates the expression of some critical transcription factors, ribosomal and spliceosomal genes. Impairments in protein synthesis and splicing mediated in part by transcription factors such as FOXC2, GATA1, contribute to impaired neuronal function and concurrence of intellectual disability and craniofacial anomalies in our patients. Our study highlights the emerging role of the spliceosome and ribosomal proteins in intellectual disability.

## Supplementary Information

**Additional file 1. Supplementary Table 1**. Dysregulated genes in POLR3B mutants patients.

**Additional file 2. Supplementary Table 2**. Details of the hub genes.

## Data Availability

The datasets used and analysed during the current study are available in GEO with accession number GSE184234.

## Abbreviations

ID: Intellectual disability
RNA: Polymerase III (Pol III)
FPKM: Fragments per kilobase of exon per million fragments mapped
PPI: Protein–protein interactome
POLR3-HLD: POLR3-related hypomyelinating leukodystrophy
NMD: Nonsense Mediated Decay
TRAF5: Tumor necrosis factor receptor-associated factor 5
TNF: Tumor necrosis factor
BBB: Blood–brain barrier
TCS: Treacher Collins syndrome
AD: Alzheimer patients
CAMs: Cell adhesion molecules
FOX: Forkhead box
NPCs: Neural progenitor cells
